# Molecular Epidemiology, Drug-Resistant Variants, and Therapeutic Implications of Hepatitis B Virus and Hepatitis D Virus Prevalence in Nigeria: A National Study

**DOI:** 10.3390/pathogens14010101

**Published:** 2025-01-20

**Authors:** Oludare ‘Sunbo Adewuyi, Muhammad Shakir Balogun, Hirono Otomaru, Alash’le Abimiku, Anthony Agbakizu Ahumibe, Elsie Ilori, Que Anh Luong, Nwando Mba, James Christopher Avong, John Olaide, Oyeladun Okunromade, Adama Ahmad, Afolabi Akinpelu, Chinwe Lucia Ochu, Babatunde Olajumoke, Haruka Abe, Chikwe Ihekweazu, Adetifa Ifedayo, Michiko Toizumi, Hiroyuki Moriuchi, Katsunori Yanagihara, Jide Idris, Lay-Myint Yoshida

**Affiliations:** 1 Graduate School of Biomedical Sciences, Nagasaki University, Nagasaki 852-8523, Japanhiromori@nagasaki-u.ac.jp (H.M.);; 2 Department of Paediatric Infectious Diseases, Institute of Tropical Medicine, Nagasaki University, Nagasaki 852-8523, Japantoizumi@nagasaki-u.ac.jp (M.T.); 3 Nigeria Centre for Disease Control and Prevention, Abuja 240102, Nigeriaoyeladun.okunromade@ncdc.gov.ng (O.O.); chinwe.ochu@ncdc.gov.ng (C.L.O.);; 4 Nigeria Field Epidemiology and Laboratory Training Programme, Abuja 900231, Nigeria; msbalogun@afenet.net; 5 African Field Epidemiology Network, Asokoro, Abuja 900231, Nigeria; 6 Institute of Human Virology, University of Maryland School of Medicine, Baltimore, MD 21201, USA; 7 Vietnam Research Station, Institute of Tropical Medicine, Nagasaki University, Nagasaki 852-8523, Japan; 8 WHO Hub for Pandemic and Epidemic Intelligence, Prinzessinnenstrasse 17-18, 10969 Berlin, Germany; 9 Foundation for Innovative New Diagnostics, 1202 Geneva, Switzerland; 10 School of Tropical Medicine and Global Health, Nagasaki University, Nagasaki 852-8523, Japan; 11 Department of Paediatrics, Graduate School of Biomedical Sciences, Nagasaki University, Nagasaki 852-8523, Japan; 12 Department of Laboratory Medicine, Graduate School of Biomedical Sciences, Nagasaki University, Nagasaki 852-8523, Japan

**Keywords:** HBV, genotypes, viral load, HDV, drug-resistant associated variants, Nigeria

## Abstract

Information on circulating HBV (sub-)genotype, variants, and hepatitis D virus (HDV) coinfection, which vary by geographical area, is crucial for the efficient control and management of HBV. We investigated the genomic characteristics of HBV (with a prevalence of 8.1%) and the prevalence of HDV in Nigeria. We utilised 777 HBV-positive samples and epidemiological data from the two-stage sampled population-based, nationally representative Nigeria HIV/AIDS Indicator and Impact Survey conducted in 2018. We assessed 732 HBV DNA-extracted samples with detectable viral loads (VLs) for (sub-)genotypes and variants by whole-genome pre-amplification, nested PCR of the *s*-and *pol*-gene, and BigDye Terminator sequencing. We conducted HDV serology. In total, 19 out of the 36 + 1 states in Nigeria had a high prevalence of HBV (≥8%), with the highest prevalence (10.4%) in the north-central geopolitical zone. Up to 33.2% (95% CI 30.0–36.6) of the participants had detectable VLs of ≥300 copies/mL. The predominant circulating HBV genotype was E with 98.4% (95% CI 97.1–99.1), followed by A with 1.6% (95% CI 0.9–2.9). Drug-resistant associated variants and immune escape variants were detected in 9.3% and 0.4%, respectively. The seroprevalence of HDV was 7.34% (95% CI 5.5–9.2). Nigeria has subtype E as the major genotype with many variants.

## 1. Introduction

Hepatitis B virus (HBV) infection, the commonest human viral hepatitis (VH), is the second leading transmittable cause of mortality on the planet [1]. Currently, HBV has a global prevalence of about 300 million people and an incidence of 1.5 million cases annually, with at least a third of the world population having been infected at different points [2,3,4]. The African region (with a prevalence of 6.1%) has been identified as one of the ‘hotspots’ for VH, with an average of one person dying of HBV infection every 2.5 min [5,6,7]. Nigeria, with a generally low complete vaccination rate (36.2 to 59.5% in healthcare workers), is reported to be one of the five countries responsible for more than 50% of global hepatitis B infections [6]. In Nigeria, the prevalence of HBV is about five times that of HIV in the 15–64-year age group: 8.1% versus 1.5%. Viral hepatitis is a disease of public health importance that receives little attention in funding, awareness, and treatment in Nigeria [8,9].

Slowly, but evidently, there is currently less transmission of HBV and more disease (resulting from previous infections of 20–40 years ago) due to epidemiological transmission in Nigeria; hence surveillance, monitoring, and effective therapy become vital as control and management strategies. The plan to eliminate VH (i.e., reduction in incidence by 90% and mortality by 65%) as a public health threat by 2030 as approved by the World Health Assembly (WHA) will prevent about 36 million infections and save about 10 million lives by that year [10]. Achieving this target requires not only preventive measures but also a more effective therapy. Available therapies [including alpha-interferon (IFN) together with six nucleos(t)ide analogues (NAs)] which only suppress HBV replication are not just expensive but have also been variously faulted, including the acquisition of drug resistance-associated variants (DRAVs) of HBV mitigating against the achievement of the WHA 2030 VH goal [11,12,13,14]. The nucleos(t)ide analogues target and suppress the reverse transcriptase (RT) in the *pol*-gene while interferon has antiviral and immune-modulating properties. Additionally, responses to therapy vary depending on the genotype and the presence or not of multiple genotype/mixed genotype infection. For instance, HBV A+D mixed genotypic infection has been documented to hold up to six times higher risk of progression to hepatocellular carcinoma (HCC) compared with HBV genotype A infection [14].

The distribution pattern of the HBV genotypes, which varies with patients and geographical location worldwide, is associated with disease progression [to liver cirrhosis, HCC, liver failure, and death], the mode of transmission, clinical outcome, and treatment response [15,16]. For instance, HBV genotypes B and C, found mainly in Asia, are predominantly transmitted vertically. In contrast, other genotypes found in other geographical areas (e.g., genotype E in West Africa and F in South America) tend to be transmitted horizontally. HBV sub-genotypes have also demonstrated defined geographical patterns and clinical outcomes. While A1 has been linked to fast progression to cirrhosis and HCC, A2 progresses more slowly. Also, A1 and B have a high response rate to interferon while C, D, and I have a low response rate in one study [16].

In addition, cases of DRAVs have been reported with the current regime of drugs. Hepatitis B viruses have a life cycle that requires an error-prone reverse transcriptase for replication. This results in tremendous genetic variation in the form of (sub-)genotypes. The error-prone HBV polymerase generates the genetic variability observed as viral quasi-species that can lead to resistance to antiviral agents. Both treatment-naïve and patients who failed treatment can have RAVs, with a large proportion of those who failed antiviral therapy acquiring resistance [17]. In some cases, acquired resistance leads to cross-resistance against other antiviral agents, limiting future options. There are four genes in the HBV genome encoding different proteins. Apart from the *pol*-gene mentioned above, naturally occurring or therapeutic-induced HBV variants with mutation can occur in other HBV open reading frames (ORFs): the *pre-S/S* region, leading to vaccine escape mutants (VEMs), viral clearance disturbance for the *preS1/preS2* region, non-response to IFN therapy for the *pre-C* region, decreased HBeAg expression for the *C* region, and tumorigenesis for *X*-gene variants [17,18,19,20].

In Nigeria (as in most LMICs), genotyping and baseline resistance testing are not routinely carried out in VH management. Also, only pockets of studies have reported that genotype E is the predominant HBV in these areas of the country. Hepatitis D virus (HDV), which causes the most severe form of viral hepatitis (VH) infection, only occurs in persons who are infected with HBV since HDV depends on HBV for its replication. Although immunisation against HBV in those not infected with the two viruses would protect against both HBV and HDV, the treatment of HBV with currently approved NAs does not influence HDV infection [21]. With no antivirals currently approved against HDV (only 48 weeks of PEG IFN alpha that only suppresses the virus in a quarter of patients is available), the proper understanding of the distribution of HDV in Nigeria is crucial. Recent WHO recommendations for chronic hepatitis B (CHB) treatment have added CHB cases with HDV coinfection. HDV affects nearly 4.5% of people globally. Up to 7.33% of the sub-Saharan African population is affected by HDV, with the national prevalence unknown in Nigeria [22,23].

For the effective management (including the choice of treatment medications, testing, and vaccine selection) and possible elimination of VH as projected, there is a need to investigate the relationship between HBV genetic variation, and drug treatment in the Nigerian population. Therefore, this study sought to elicit the distinct genetic characterisation of HBV circulating in Nigeria including clinically relevant variants that may influence therapies to guide HBV precision management and HDV prevalence in Nigeria.

## 2. Materials and Methods

### 2.1. Study Design and Samples

**Figure 1:**
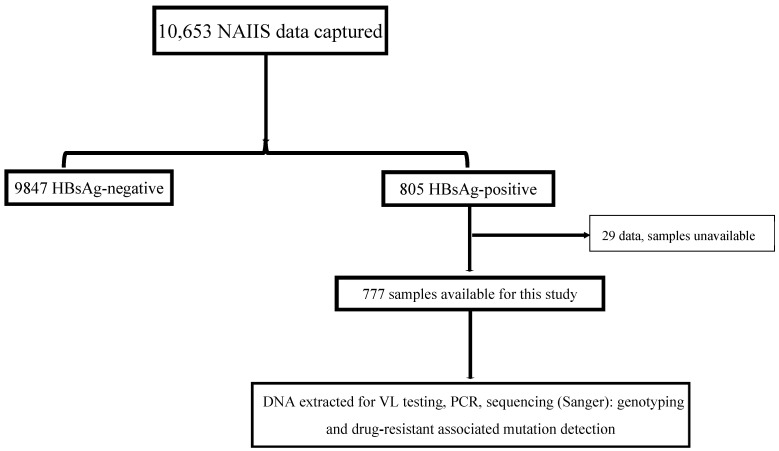
Study flow chart of the national HBV study in Nigeria, 2024. NAIIS, Nigeria HIV/AIDS Indicator and Impact Survey; HBsAg, hepatitis B surface antigen; VL, viral load.

This cross-sectional, nationally representative study was nested in the recent Nigeria HIV/AIDS Indicator and Impact Survey (NAIIS) study. NAIIS was a two-stage, household cluster study of 15-to-64-year-olds. The survey sampled enumeration areas (EAs) followed by households. The EAs were mutually exclusive and all households in Nigeria had an equal chance of being included in the survey. The first stage of sampling selected 4035 EAs using a probability proportional to size method. The 4035 EAs were stratified by Nigeria’s 36 states and the FCT. The second stage selected a random sample of households within each EA using an equal probability method [7]. All plasma samples and epidemiological data used for this study were retrieved from the biorepository of the NAIIS study at the National Reference Laboratory (NRL) of the Nigeria Centre for Disease Control and Prevention (NCDC) in Gaduwa, Abuja. The 10,653 community participants’ data captured for the VH arm of NAIIS yielded a national prevalence of 8.1%. In total, 777 HBV-positive plasma samples were received from the NCDC NRL for this study (Figure 1). Up to 94.2% (732) of these samples were included in downstream laboratory analyses.

From the blood samples collected, HBV screening using the Determine™ HBsAg test kit (Abbott Inc., Chicago, IL, USA) was conducted during NAIIS. The rest of the specimens were processed into plasma aliquots and dried blood spots (DBSs) and stored appropriately [7]. Subjects included in this study were 15–64-year-olds with positive HBsAg tested via the Determine™ HBsAg kit. We excluded participants with inadequate plasma samples (≤200 μL).

### 2.2. Hepatitis D Virus ELISA

The human hepatitis D virus (HDV) antibody (IgG) ELISA Kit (CUSABIO TECHNOLOGY LLC, Houston, USA, catalogue number CSB-E04809h) was used to perform HDV serology on 777 HBV samples (763 plasma and 14 DBSs), according to the manufacturer’s instructions.

### 2.3. Nucleic Acid Extraction from HBsAg-Positive Blood Samples and Quantitative Polymerase Chain Reaction

The HBV DNA extraction was performed on 50 μL of all available HBV plasma samples (763) using the SMITEST EX-R&D kit (catalogue number GS-J0201), MBL, Japan, as per the manufacturer’s instructions [24]. The DBS DNA Isolation Kit by Norgen Biotek, Ontario, Canada (catalogue number 36000) was used to extract HBV DNA from 14 DBS-stored samples, according to the manufacturer [25]. The final elution volume of extracted HBV DNA was 20 μL, all carried out at the NCDC NRL, Abuja, Nigeria. The HBV DNA levels were assessed by a real-time PCR assay using the StepOnePlus real-time PCR system, California, USA (the protocol number for the MicroAmp™ Fast 8-Tube Strip is 4323032) as previously described, with a lower limit of detection of 2.3 IU/mL [26].

### 2.4. Amplification of HBV DNA for Sequencing, Sequencing of S- and Pol- Genes, Alignment, and Phylogenetic Analysis, and Mutational Analysis

Please refer to supplementary data for details on these methods.

### 2.5. Data Analysis

Frequencies and percentages were used to describe the characteristics of the samples. The Chi-Square (χ2) test was used to test the differences between groups (or Fishers’ exact test if the cell count was ≤ 5). The obtained values were considered statistically significant at *p* ≤ 0.05. We employed logistic regression for multivariate analysis to identify factors associated with variants. In the multivariate analysis, we calculated adjusted odds ratios employing a backward stepwise selection. Phylogenetic trees were produced using Geneious Prime software version 2024.0.7 and adjusted visually with the iTOL tool version 7. The trees were aligned with the MAFFT algorithm.

## 3. Results

### 3.1. Characteristics of HBV-Positive Respondents and HDV Seroprevalence

**Figure 2:**
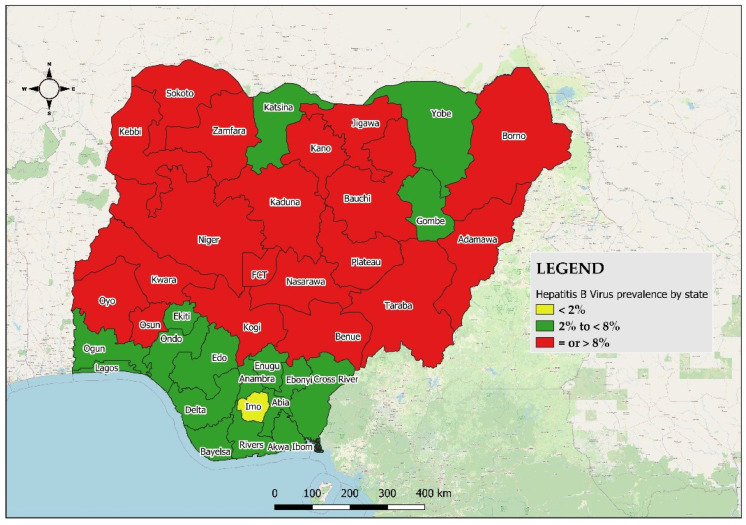
Geographical distribution of all HBV samples by state (and the FCT) according to prevalence group in Nigeria, 2024.

Out of the 805 subjects that were HBsAg^+^ in NAIIS, 777 samples were available at the biorepository for our study. The median age of HBV-positive respondents was 32.5 years (range: 15, 64) with the 25–29-year age group having the highest proportion of HBV seroprevalence [male = 19.5% (16.0–23.6) %; female = 17.0% (13.4–21.1) %] (Figure S1). A total of 19 states out of the 36 + 1 states had an HBV prevalence of ≥8%, whereas Imo State had <2% (Figure 2 and Table S2). The northcentral geopolitical zone had the highest prevalence, 10.4% (*p*-value <0.001). Up to 66.8% (63.4–70.0) of HBV positives had a detectable viral load (VL) of <300 c/mL, followed by 16.7% (14.3–19.5) with 300–9999 c/mL, and 16.5% (14.0–19.3) with ≥10,000 c/mL. Multivariate analysis revealed that being in the 55–59-year age group, a female, and living in the southeast and southsouth geopolitical zones are relatively lower risks of being infected with HBV in Nigeria (Table 1). The seroprevalence of HDV among all the 777 HBV-positive subjects was 7.34% [CI: (5.5–9.2)]. Of the 57 HDV-positive persons, males were 40.4% (28.3–53.7); females and the 20–24- and 30–34-year age groups had the highest frequency [17.5% (CI: 9.6–29.9) each]. Up to 19.3% (11, CI: 10.9–31.9) of HDV/HBV-coinfected individuals had HBV VLs of ≥10,000 c/mL.

### 3.2. Circulating (Sub-)Genotypes in Nigeria

**Figure 3:**
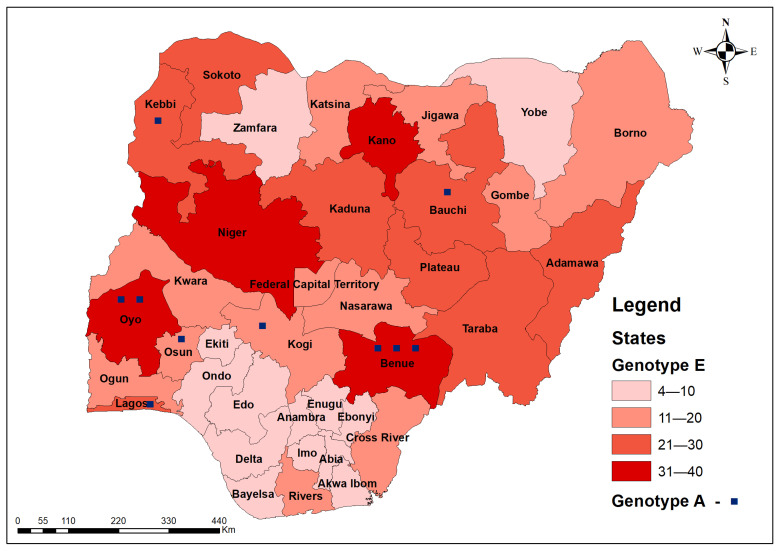
Geospatial distribution of all 626 *s*- and *pol*-genes sequenced HBV genotypes in Nigeria (heat map showing hotspots) of HBV genotypes in Nigeria, 2024.

**Figure 4:**
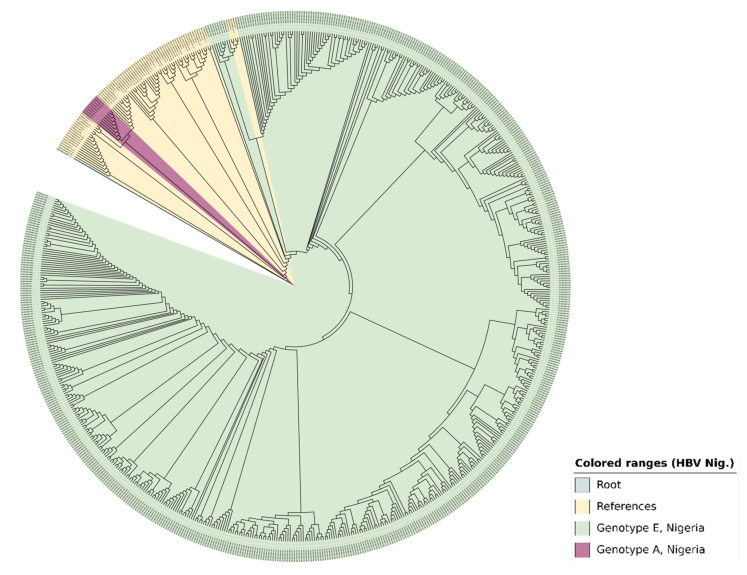
The phylogenetic tree generated with the *s*-gene (samples with *F* and *R* reads) using Geneious Prime and enhanced visually by iTOL displays the identified 519 HBV genotypes in Nigeria, 2024. An MAFFT alignment was carried out and the dendrogram was constructed via the neighbour-joining method. The outgroup was an HBV sequence from a gorilla with accession number AJ131567. There were 76 reference sequences from the GenBank representing all HBV (sub)types (A—J).

Two genotypes were detected from 626 out of 777 analysed samples (80.6%) of successfully sequenced *s*- and *pol*-genes: genotypes E, 98.4% (97.1–99.1), and A, 1.6% (0.9–2.9) with A2, A3, C2, D1, D3, E, F4, and G subtypes (Figure 3 shows the hotspots of genotypes A and E for all samples while Figure 4 shows the phylogenetic tree for samples with *F* and *R* reads using the HBV *s*-gene). The density of genotype E is highest (>20) in the following states: Kano (35), Oyo (33), Niger (32), Benue (32), Kaduna (28), Kebbi (26), Taraba (25), Lagos (25), Bauchi (24), Sokoto (22), Plateau (22), and Adamawa (21). Genotype A was detected in Benue (3), Oyo (2), Bauchi (1), Kebbi (1), Kogi, Lagos (1), and Osun (1) states.

### 3.3. Therapeutically Important Variants in Nigeria

We detected 72 (9.3%) DRAVs and 3 (0.4%) IEVs, altogether accounting for 9.7% (7.8–12.0) out of 777 samples. The DRAVs were against four (Adefovir, Entecavir, Lamivudine, and Telbivudine) out of five nucleos(t)ide analogues with no resistance against Tenofovir. The mutations include 80X, 169X, 173X, 180X, 181X, 184X, 202X, and 204X (DRAVs) and 137X and 145K (IEVs) (Table 2 and Figure S2). The 169X and 173X mutations have the widest spread (23 states; Table S4 and Figure S2). Two IEVs (137X and 145K) were identified (summary in Table 2). The significance of some of the identified variants is yet to be understood. Only 23.3% [14.9–34.5] of the 73 samples with detected RAVs have one mutation (the remaining 76.7% [65.5–85.1] have >1 RAVs). Residence in the southeast (aOR = 2.6; 95% CI: 1.1–6.4, *p* = 0.038) and being in the age groups 30–34 years (aOR = 3.5; 95% CI: 1.3–10.3, *p* = 0.015) and 55–59 years (aOR = 6.7; 95% CI: 1.3–37.5) are predictors of RAVs (Table 3).

## 4. Discussion

This is the first nationwide, population-based study in Nigeria genetically characterising HBV. We found a high prevalence of HBV (≥8%) in over 50% of the Nigerian states and the northcentral geopolitical zone, with the highest prevalence in the 25–29-year-old age group [27]. This shows that HBV infection remains a public health crisis in most parts of the country, with attendant implications such as sustained transmission, vaccination challenges, disease progression, and an unabated management burden. The northcentral geopolitical zone has been known to have a relatively low vaccine coverage. Other potential factors, such as limited access to healthcare facilities because of being in a hard-to-reach area or on account of population displacement, and cultural practices may play a role. Future research in these settings will be important to determine the risk factors for future preventive measures. A third of the HBV positives had a VL of ≥300 c/mL and half of those had a VL of ≥10,000 c/mL. The chances of disease progression are lower at VLs of <300 c/mL while those with a VL of 10,000 or more should be considered for therapy according to several guidelines [28,29]. In essence, without the required awareness, testing, and therapy, up to 16.7% of our study participants may continue to transmit HBV while they remain at risk of the sequelae of the infection. Like previous studies, our study shows that being male is a risk factor for HBV infection positivity. Residents of the southeast geopolitical zone have a lower risk of HBV infection. This may not be unrelated to the lower prevalence of HBV observed throughout the five states in this region. Also, those in the 55–59-year age group have a lower risk of HBV infection. The average Nigerian is in the younger age group; hence, most infections may be associated with being in these younger, more sexually active, and other HBV risk-prone age groups.

Although no previous nationwide, community-based HBV studies have been conducted in Nigeria, past studies revealed genotype E as the main circulating HBV subtype [30]. We detected two HBV subtypes, A and E, with genotype E being the predominant genotype with most of the burden in ten northern states. Apart from being the predominant strain in West and Central Africa, it has been demonstrated that African emigrants to Europe and other parts of the world who are HBV carriers have genotype E [31,32,33]. Genotype E has been associated with higher VL, HBeAg positivity, chronicity, and poor response to interferon relative to other genotypes (apart from genotype C) [31,34,35]. The efficacy of NAs to genotype E is unclear since it was not considered in producing current treatment guidelines [32]. Genotype E has a low nucleotide divergence (only two lineages reported and also being related to genotype D and HBV chimpanzee strains), indicating recent advancement to humans [32]. Although its main mode of transmission is horizontal, perinatal transmission has been reported and is important in our study area. Conversely, genotype A, which has been reported to spread mainly horizontally (A1, horizontally; A2, vertically) with persistent HBeAg positivity, responds best to interferon with less aggressive clinical outcomes compared with other genotypes. Genotype A has seven subtypes which are almost equally susceptible to the NAs when compared to other genotypes [36,37,38,39]. The finding of genotype E as the predominant circulating subtype offers an opportunity to have a tailored management approach in Nigeria and the African subregion by starting with an evaluation of the efficacy of current antivirals (to potentially develop drugs specifically targeting HBV genotype E since this genotype was not considered in the generation of current treatment guidelines) while sustaining HBV (sub-)genotype surveillance.

Our study is the first national study to report the HBV variant in Nigeria. The national prevalence of the detected HBV DRAVs was 9.3% and that of IEVs was 0.4 (total HBV variant prevalence, 9.7%), with more than 75% of these being HBV multidrug-resistant [40]. Only a country-wide study of this magnitude can provide such valuable information. Disease progression and the lack of viral suppression have been attributed to the presence of DRAVs [41]. The NAs target and suppress the reverse transcriptase (RT) on the *pol* ORF by mimicking natural nucleosides during viral replication. This inhibits the HBV DNA polymerase activity, leading to the suppression of HBV replication. Naturally occurring or therapeutically induced HBV DRAVs can be clinically relevant. The primary HBV mutations detected in our study include DRAVs 169X, 181X, 184X, 202X, and 204X, where the pivotal codons confer direct resistance on NAs, while 80X, 173X, and 180X are compensatory: the variants compensate for fitness loss associated with primary mutation [42,43,44]. It is a concern for treatment options that all the HBV DRAVs detected were resistant to four out of the five NAs on the database considered; only tenofovir remains a susceptible NA and remains indisputably the recommended antiviral of first choice in the country. This study revealed only two types of IEVs (137X and 145K). The two detected IEVs are unknown mutations on a rated position found in the “a” determinant area with potentials for immune escape where vaccinated persons still get infected with HBV [45]. The mutation of HBsAg at positions 137–147 can alter the conformational epitope within the “a” determinant, preventing it from being detected by neutralising anti-HBs (hence undetected by serological investigations too: diagnostic failure) [46]. Since both genotypes A and E circulating in Nigeria have a common conserved “a” determinant area (aa 99–160) in their HBsAg, which (subtype A2) vaccines target, and we recorded a national IEV prevalence of 0.4%, the current HBV vaccine is expected to remain effective in Nigeria, which may in part explain the few IEVs detected in Nigeria.

The emergence of HBV variants can be influenced by host factors (host immune pressure, VL, and coinfections), viral factors (fitness and errors during replication) and/or external factors (vaccination, prior exposure to NAs, and NA genetic barriers). Residence in the southeastern geopolitical zone was associated with having an HBV variant [47,48]. This is similar to other studies globally, reflecting geographical or regional significant differences in HBV variants within a country or continent. In Nigeria, more long-term therapeutic use of herbal antiviral agents has been reported in the SE [49]. The cross-resistance of some of these agents may contribute to the development of HBV variants. Although high pre-treatment VL is documented to be associated with HBV variants [50], there was no significant association in our study. On account of immunity, HBV variants are expected to be associated with the extremes of age [51]. The 55–59-year age group fits into this, but the 30–34-year age group does not. There may be other factors like coinfection, VL, genotype, vaccination status, and previous NA exposure at play here that require further investigation.

HDV-HBV coinfection presents the most severe VH disease with faster progression and poorer prognosis compared with mono-infective VH. The HDV seroprevalence from this study was 7.34% using the antigenically distinct Cusabio ELISA kit that determined the HDV antibody. This brings the burden of HDV in Nigeria to about 1.4 million people considering the current population. Recent pockets of HDV prevalence studies in Nigeria were commonly conducted in health facilities. Such hospital-based studies have demonstrated varying prevalence ranging from 9 to 19% [52,53,54,55]. However, our finding is similar to the population seroprevalence of HDV in West Africa (7.33%), but well above the global average of 4.5% [56]. This is the first time a national prevalence of HDV has been determined in a house-to-house-based study in Nigeria. This implies that the contribution of HDV to the burden of hepatic disease is quite significant in Nigeria. Understanding the burden of HDV will provide essential information to enhance the surveillance and control of HDV infection in Nigeria. More studies to determine the circulating genotypes of HDV and to identify the risk factors for HDV infection and its genotype will be crucial in guiding HDV management in Nigeria.

Recent WHO CHB new treatment guidelines recommend the treatment of CHB cases with (i) evidence of significant fibrosis, (ii) HBV DNA >2000 IU/mL and an ALT level above the upper limit of normal, (iii) the presence of coinfections (such as HIV, hepatitis D, or hepatitis C), and (iv) those with persistently abnormal ALT levels. However, practical application is limited due to the high price of the drugs. From our findings, the optimal therapeutic management of HBV in Nigeria should include the following. A clinical review of how the dominant circulating genotype E interacts with the currently approved NAs is advised; meanwhile, tenofovir should be the first line of medication in qualified patients. Baseline genotyping and RAVs assessment, though beneficial in the management of HBV, are costly. Interventions by the government and other stakeholders are necessary in order to subsidise HBV management. Evidence from this study suggests that the use of the current HBV (subtype A2) vaccine remains beneficial in Nigeria. Deployment or accelerated HBV control approaches including awareness creation, affordable, accessible investigations (especially genotyping), and targeted management in addition to current good clinical practices are crucial to VH elimination in Nigeria and the world as scheduled by the WHO, with a focus on the northcentral and the southeastern geopolitical zones.

We recognise the following limitations. Those with known HIV status may have refused to participate in the original NAIIS study. Since HIV-positive individuals have a higher risk of HBV infection, there is a possibility that our study result may be underestimating the real HBV burden in Nigeria. Adding biomarker investigations would have complemented the outcome of this study but we worked with limited samples in the available timeframe. Also, conducting next-generation sequencing would provide more robust data in detecting novel (sub-)genotypes and variants since Sanger sequencing detects only one of the dominant subtypes [44]. We hope to be able to carry this out in the future.

## 5. Conclusions

This first nationwide, population-based HBV study in Nigeria identified HBV genotype E as the predominant circulating subtype, national HBV drug-resistant associated variants, immune escape variants, and HDV prevalences of 9.3, 0.4, and 7.34%, respectively. Sustained national and regional surveillance of HBV variants is crucial to understanding their trend, impacts on HBV vaccination, and management. Our study also demonstrated the considerable role of conducting baseline viral genomic investigations (routine baseline genotyping, DRAV testing, and VL testing) in addition to the usual biochemical workup for HBV management in Nigeria and the region. These will guide in enhancing a personalised approach to HBV infection treatment, limiting the incidence of treatment failure in a clime where coinfection and comorbidity are common. With a significant national HDV prevalence of 7.34%, HBV-positive patients should be screened for HDV.

## Figures and Tables

**Table 1:** Multivariate factors associated with HBsAg positivity (model *p* < 0.001; N = 8767).

Risk Factor	OR	aOR	*p*-Value	95% CI	
Age group					
15–19	1	1			
20–24	1.1609	1.4336	0.132	0.8971	2.2909
25–29	1.1863	1.2898	0.282	0.8114	2.0503
30–34	1.0640	1.1155	0.653	0.6922	1.7978
35–39	1.2203	1.4099	0.159	0.8742	2.2738
40–44	0.9966	0.9739	0.918	0.5877	1.6141
45–49	0.6340	0.6772	0.169	0.3885	1.1805
50–54	0.6554	0.6689	0.163	0.3805	1.1762
55–59	0.3119	0.3699	**0.008**	0.1775	0.7712
60–64	0.5203	0.6933	0.248	0.3724	1.2908
Sex					
Male	1	1			
Female	0.6068	0.6298	**<0.001**	0.5218	0.7602
Geopolitical zone					
NW	1	1			
NE	0.9539	0.8727	0.328	0.6645	1.146
NC	1.1501	1.1522	0.296	0.8833	1.503
SE	0.3688	0.3558	**<0.001**	0.2326	0.544
SS	0.4182	0.4086	**<0.001**	0.2822	0.592
SW	0.8830	0.8644	0.363	0.6314	1.183
Marital status					
Never married	1	1			
Married (or live-in)	0.8742	1.0356	0.794	0.7967	1.3462
Divorced (/Sep./Wid.)	0.7035	1.2714	0.198	0.8819	1.8329
Education					
None	1	1			
Primary	0.9930	1.1001	0.493	0.8372	1.4457
Secondary	1.2028	1.2026	0.183	0.9165	1.5779
Tertiary	1.2307	1.2653	0.167	0.9061	1.7667
Other	1.2628	0.8525	0.393	0.5912	1.2291
Wealth quintile					
Lowest	1	1			
Second	0.9773	1.0564	0.669	0.8212	1.3590
Middle	0.7804	0.9313	0.609	0.7090	1.2232
Fourth	0.7709	0.8887	0.440	0.6587	1.1989
Highest	0.6722	0.8326	0.287	0.5941	1.1668
Coitarche age group					
0–14	1	1			
15–19	0.9957	0.9440	0.704	0.7009	1.2714
20–24	1.1727	1.0302	0.857	0.7453	1.4241
≥25	1.3095	1.0905	0.646	0.7539	1.5775

We conducted a backward stepwise selection (with a probability of entry *p* <  0.25 and a probability of removal *p*  <  0.3) with education and coitarche age group as forced variables while avoiding colinear variables. The model also adjusted for potential confounders including geopolitical zone, marital status, and wealth quintile. OR = odds ratio; aOR = adjusted odds ratio; *p* = *p*-value  <  0.05; CI = 95% confidence interval, significant *p*-values are boldly written.

**Table 2:** Clinically relevant DRAVs and IEVs in Nigeria.

Sample IDs	Mutation	Sample Freq.	Resistance					Remark(s)
			Lamivudine	Adefovir	Entecavir	Tenofovir	Telbivudine	
39, 54, 59, 60, 62, 121, 141, 144, 150, 189, 192, 205, 206, 209, 212, 216, 224, 378, 444, 447, 452, 470, 477, 522, 524, 527, 529, 533, 587, 632, 658, 723, 730, 733, 739, 751,	173X	32	Yes					
39, 54, 59, 60, 62, 69, 104, 108, 121, 141, 144, 205, 216, 527, 189, 192, 378, 444, 447, 452, 477, 468, 529, 587, 632, 645, 705, 723, 730, 733, 739, 751,	181X	36	Yes	Yes *			Yes	Unknown mutation on rated position
39, 47, 48, 50, 51, 52, 53, 59, 60, 61, 76, 79, 104, 108, 121, 141, 144, 205, 216, 527, 189, 444, 452, 470, 477, 529, 587, 632, 644, 658, 674, 705, 723, 730, 733, 739, 751,	180X	38	Yes		Yes *			Compensatory mutation
39, 54, 59, 60, 141, 160, 175, 189, 192, 205, 206, 209, 212, 224, 226, 296, 343, 354, 358, 378, 447, 452, 529, 591, 632, 658, 723, 730, 739, 751	169X	30			Yes *			Compensatory mutation: 169 is a rated position
39, 54, 60, 62, 76, 79, 121, 141, 144, 150, 189, 192, 205, 209, 360, 435, 452,529, 632, 658, 730, 739, 751,	184X	23			Yes *			Unknown mutation on rated position
43, 47, 48, 50, 51, 53, 55, 57, 59, 122, 449, 479, 501, 645, 686,	80X	15	Yes				Yes	Compensatory mutation
39, 39, 47, 48, 50, 51, 52, 60, 62, 72, 76, 82, 104, 144, 209, 378, 470, 568, 468,529, 587, 632, 644, 658, 733, 739,	204X	26	Yes		Yes		Yes	
39, 50, 51, 52, 60, 62, 76, 108, 144, 150, 378, 452, 470, 529, 587, 632, 733, 739, 751,	202X	19			Yes			
444	137W	1						IEVs
444	137Y	1						
444	145K	1						

DRAVs, drug-resistant associated variants; IEVs, immune escape variants; IDs, identification numbers. Up to 76.7% (65.5–85.1) of the detected variants in Nigeria are HBV multidrug-resistant [i.e., resistant to more than one nucleos(t)ide analogues]. * Some of the variants are unknown mutations on rated positions.

**Table 3:** Risk factors for HBV variants in Nigeria, 2024.

Variable	No DRAVs	DRAVs	OR	aOR	*p*-Value	95% CI of aOR	
Sex (n = 774)							
Male	370	45	1	1			
Female	330	30	0.74747	0.82664	0.474	0.49065	1.39272
Age group (n = 775)							
15–19	73	5	1	1			
20–24	104	8	1.12308	1.33883	0.581	0.42814	4.54738
25–29	132	10	1.10606	1.15761	0.744	0.39068	3.73013
30–34	96	21	3.19375	3.46681	**0.015**	1.28034	10.3278
35–39	118	10	1.23729	1.52356	0.428	0.50935	4.90478
40–44	77	8	1.51688	1.61375	0.343	0.54139	5.83818
45–49	36	5	2.02778	2.22624	0.202	0.62324	9.34998
50–54	34	3	1.28824	1.51776	0.503	0.3676	7.68361
55–59	9	3	4.86667	6.66269	**0.023**	1.3124	37.4784
60–64	21	2	1.39048	1.41425	0.652	0.25794	8.70352
VL cat.							
<300	470	49	1	1			
300–99999	117	13	1.06576	0.77086	0.451	0.39211	1.51546
>10000	115	13	1.0843	0.74173	0.393	0.37362	1.47253
HBV/HDV							
No	652	68	1	1			
Yes	50	7	1.34235	1.17434	0.718	0.49163	2.8051
GP zone							
NW	171	19	1	1			
NE	121	8	0.59504	0.58627	0.237	0.24217	1.41929
NC	180	17	0.85	0.84031	0.636	0.40908	1.72614
SE	38	10	2.36842	2.5869	**0.038**	1.05432	6.34729
SS	65	7	0.96923	0.94019	0.898	0.36476	2.42342
SW	125	14	1.008	1.09593	0.813	0.51235	2.34421

The model controlled for viral load, HBV/HDV co-infection, and geopolitical zone. OR = odds ratio; aOR = adjusted odds ratio; *p* = *p*-value  <  0.05; CI = 95% confidence interval; significant *p*-values are boldly written; DRAVs = drug resistance-associated variants; VL cat. = viral load category; GP zone = geopolitical zone (NW = northwest; NE = northeast; NC = northcentral; SE = southeast; SS = southsouth; and SW = southwest geopolitical zones).

## Data Availability

Although data are not publicly available due to ethical considerations, they can be made available upon reasonable request.

## Supplementary Materials

The following supporting information can be downloaded at: https://www.mdpi.com/article/10.3390/pathogens14010101/s1, Table S1: Primer sequences used for HBV DNA sequencing. Table S2: Geographical distribution of HBV by state (and the FCT) according to prevalence group in Nigeria, 2024. Table S3: List of therapeutically relevant HBV variants from the literature. Table S4: Geographical distribution of HBV variants in Nigeria, 2024. Figure S1: Age–sex population pyramid of HBV+ in Nigeria, 2024. Figure S2: Distribution of HBV variants by state in Nigeria, 2024. Refs. [57,58,59,60,61,62,63,64,65,66,67] are cited in the supplementary materials.
