# Supplementary material for: Molecular Epidemiology, Drug-Resistant Variants, and Therapeutic Implications of Hepatitis B Virus and Hepatitis D Virus Prevalence in Nigeria: A National Study
Source: Pathogens. 2025 Jan 20;14(1):101. doi: 10.3390/pathogens14010101 (PMC11768191; doi:10.3390/pathogens14010101)
Supplement: Supplementary file 1 [file pathogens-14-00101-s001.zip › pathogens-3417646-supplementary.pdf]

Molecular epidemiology, variants, and therapeutic implications of hepatitis B virus and hepatitis D virus prevalence in Nigeria: a national study

Oludare 'Sunbo Adewuyi, Muhammad Shakir Balogun, Hirono Otomaru, Alash'le Abimiku, Anthony Agbakizua Ahumibe, Elsie Ilori, Que Anh Luong, Nwando Mba, James Christopher Avong, John Olaide, Oyeladun Okunromade, Adama Ahmad, Afolabi Akinpelu, Chinwe Ochu, Babatunde Olajumoke, Haruka Abe1, Chikwe Ihekweazu, Adetifa Ifedayo, Michiko Toizumi, Jide Idris, Lay-Myint Yoshida.

Table of contents

Methods 3

Amplification of HBV DNA for sequencing 3

Sequencing of *S*- and *pol*- genes 3

Alignment and phylogenetic analysis 3

Mutational analysis 4

Supplementary Tables 5

Supplementary Figures 10

References 13

Methods

Amplification of HBV DNA for sequencing

Conducting the whole genome pre-amplification (WGA) was conducted to increase the DNA copy number in the sample to increase the chances of successful amplification by increasing the amount of starting material for subsequent experiments. The HBV WGA was achieved using the KOD One PCR Master Mix (Toyobo) together with the primer set HBPr108 and HBPr109 (Table S1) on the automated ABI 9902 Veriti cycler (Applied Biosystems®) [1]. Using half reaction volumes on account of the availability of a restricted quantity of DNA, a 25μL reaction mix comprising 12.5μL of KOD Master Mix (MM), 1.25μL each of the primers, 5μL of HBV DNA, and 5μL of sterile water was conducted. The reaction included: denaturation at one cycle of 98°C, then 55°C and 68°C, each for 30s; annealing at 40 cycles of 98°C for 10s, followed by 55°C for 5s and then 68°C for 17s while elongation was at one cycle of 98°C for 10s, then 55°C for 1m and the 68°C for 5m.

Sequencing of *S*- and *pol*- genes

From the amplified products of the HBV genome, the sequences of the HBV surface gene and the reverse transcriptase portion of the polymerase gene were processed in a nested PCR. While the primer set HBPr1 and HBPr135 with KOD MM were used for the *s*-gene nested reaction (producing a 1.2kb product) with similar conditions as the whole genome sequencing (with only an adjustment of 2μL amplified DNA and 8μL of water), the primer set YMDDF2 and YMDDR2 together with Takara DNA polymerase was employed for the generation of the 409 bp amplicons *pol*-gene nested PCR (Table S1). The *pol*-gene PCR contained an adjusted 2μL of amplified DNA products because of the limited volume of samples with other components as described before [2]. The conditions for the *pol*-gene reaction were denaturation at one cycle of 94°C for 2m, then 55°C for 1m and 72°C for 1m; annealing at 35 cycles of 94°C for 30s, followed by 55°C for 30s and then 72°C for 1m while elongation was at one cycle of 94°C for 1m, then 55°C for 2m and the 72°C for 3m. The PCR products (amplicons) were visualised on a 1.25% agarose gel stained for HBV DNA/*s*-gene products and 2.0% agarose gel for products of *pol*-gene PCR with ethidium bromide.

ExoSAP-IT™ Express PCR Product Cleanup kit was applied to enzymatically purify the nested amplicons [3]. Subsequently, amplicons were sequenced using the BigDye Terminator platform after appropriate preparation of the forward (F) and reverse (R) sample products for sequencing (to increase the confidence of base calling).

Alignment and phylogenetic analysis

The sequenced data were processed (the reads were trimmed, F and R reads merged into consensus sequence, aligned using the MAFFT algorithm) and the dendrogram was plotted with the Geneious Prime software version 2024.0.7. A gorilla HBV sequence was used as an outgroup and the iTOL tool (https://itol.embl.de/itol.cgi) was utilised to adjust the generated phylogenetic tree visually.

Mutational analysis

To prevent the inclusion of polymorphic mutants and focus on the therapeutically relevant variants, a list of known relevant variants was compiled from reviewed literature for this project (Table S3). The sequences/reads were assessed for mutation (DRAVs and VEVs) using the Geno2pheno apparatus (https://hbv.geno2pheno.org/) and the NCBI website (https://www.ncbi.nlm.nih.gov/projects/genotyping/formpage.cgi) while also confirming the (sub)genotypes in the process.

Table S1: Primer sequences used for HBV DNA sequencing

Primer name	Sequence (5' – 3'), Target genes	Polarity❄
HBPr108	TTTTTCACCTCTGCCTAATC, *X* & *Core* genes	S
HBPr109	AAAAAGTTGCATGGTGCTGG, *X* & *Core* genes	AS
HBPr1	GGGTCACCATATTCTTGGG, *pol* gene	S
HBPr135	CA(A/G)AGACAAAAGAAAATTGG, *s* & *pol* genes	AS
YMDDF2	CTGTATTCCCATCCCATCATC, *s* & *pol* (RT region) genes	S
YMDDR2	GACCCACAATTCGTTGACATAC, *s* & *pol* (RT region) genes	AS

❄S: Sense primer; AS: antisense primer

Table S2: Geographical distribution of HBV by states (and the FCT) according to prevalence group in Nigeria, 2024

State	HBV+	HBV-	Total	Prev. (%)
Imo	6	315	321	1.9
A-Ibom	13	452	465	**2.8**
Delta	9	308	317	**2.8**
Abia	9	273	282	**3.2**
Bayelsa	5	144	149	**3.4**
Anambra	12	302	314	**3.8**
Enugu	11	264	275	**4.0**
Edo	12	271	283	**4.2**
Rivers	19	389	408	**4.7**
Ebonyi	12	188	200	**6.0**
Ogun	14	205	219	**6.4**
Lagos	33	478	511	**6.5**
Ondo	15	216	231	**6.5**
Katsina	23	324	347	**6.6**
Gombe	18	237	255	**7.1**
C-River	18	235	253	**7.1**
Yobe	10	128	138	**7.2**
Ekiti	13	157	170	**7.6**
Kogi	19	218	237	**8.0**
Osun	18	201	219	**8.2**
Kaduna	36	396	432	**8.3**
Jigawa	25	273	298	**8.4**
Borno	18	189	207	**8.7**
Taraba	32	325	357	**9.0**
Nasarawa	23	231	254	**9.1**
Kano	44	439	483	**9.1**
Bauchi	30	292	322	**9.3**
Zamfara	14	136	150	**9.3**
Sokoto	22	211	233	**9.4**
Plateau	29	263	292	**9.9**
Adamawa	27	230	257	**10.5**
FCT	23	192	215	**10.7**
Benue	55	438	493	**11.2**
Kwara	17	134	151	**11.3**
Niger	38	280	318	**11.9**
Oyo	48	322	370	**13.0**
Kebbi	35	191	226	**15.5**

Prev.= prevalence

Table S3: List of therapeutically relevant HBV variants from the literature [4–11]

HBV mutation	Affected ORF	Remark
Wild type	-	S to all; no mutations
		DRAVs to any of ….
rtA181T/V	pol-gene	R to ADV
rtA194T	pol-gene	R to TDF
rM204I	pol-gene	R to 3TC, & TBV
rM204V	pol-gene	R to ETV, TBV & 3TC
rtM250V	pol-gene	R to ETV
rtI169T	pol-gene	R to 3TC, TBV & ETV
rtA181T/V	pol-gene	R to 3TC
rtV173L	pol-gene	R to TBV, & 3TC
rtA194T	pol-gene	R to TDF
rtV173L	pol-gene	R to 3TC, & ETV
rtL180M	pol-gene	R to ETV, TBV & 3TC
rtN236T	pol-gene	R to ADV
rtQ215S	pol-gene	R to 3TC
rt V191I	pol-gene	R to 3TC
rt I233V	pol-gene	R to ADV
rt S213T	pol-gene	R to ADV
rtV214P	pol-gene	R to ADV
rtN236T	pol-gene	R to TDF
rtS202I/G	pol-gene	R to ETV, & 3TC (184G is R to TBV)
tL80V/I	pol-gene	R to 3TC
rtT184S/G	pol-gene	R to ETV, & 3TC (184G is R to TBV)
rtP177G	pol-gene	R to TDF
rtF249A	pol-gene	R to TDF
rt V207L	pol-/s-genes	DRAV + IEVs
T128N	s-gene	IEVs
V142I	s-gene	
W196S/L	s-gene	
I195M	s-gene	
E164D	s-gene	
I195M	s-gene	
M198I	s-gene	
W199S/L	s-gene	
W182stop	s-gene	
S204H	s-gene	
Y206H	s-gene	
P120T	s-gene	
M133I	s-gene	
D144E	s-gene	
T116N, P120S/E, I/T126A/N/I/S, Q129H/R, M133L, K141E, P142S, D144A/E, G145R/A	s-gene	IEVs (all VEVs)
Y100S, Q101R, P105R, T115N, T116N, G119R, P120L, R122P, T123N, C124R/Y, T126I/S, P127H/L, Q129P/R, M133T Y134C, S136P, C139R, T140I, K141E, S143L, D144A, G145R/A, S167L, R169H, S174N, L175S, V177A, Q181STOP	s-gene	IEVs (All OBIs)

C1653T, T1753C, A1762T, G1764A, G1896A, G1899A, A1762T, G1764A, G1862T, G1896A, Pre-S deletion	core-gene	G1896A, G1899A,= HBe seroconversion ^ HCC, Pre-S deletion HCC
3'-HBx deletion	x-gene	
C1653T, T1753C, A1762T, G1764A	x-gene	HCC

S= Sensitive; I= Intermediate/reduced susceptibility); R= Resistant

Lamivudine= 3TC; ETV, Entecavir; TDF= Tenofovir disoproxil fumarate; TAF= Tenofovir alafenamide; ADV= Adefovir; TBV: Telbivudine; DRAVs= drug resistance associated variants, IEVs= immune escape variants; VEVs= vaccine escape variants; OBIs= occult blood infections; HCC= hepatocellular carcinoma.

Table S4: Geographical distribution of HBV variants in Nigeria, 2024

Mutation	States	GP-zone
137W	Abia state	Southeast
137Y	Abia state	Southeast
145K	Abia state	Southeast
169X	Abia, Bauchi, Benue, Borno, Cross River, Delta, Ebonyi, Edo, FCT, Imo, Jigawa, Kaduna, Kano, Katsina, Kebbi, Kwara, Lagos, Nasarawa, Niger, Ogun, Oyo, Plateau, Sokoto states	Southeast, Northeast, Northcentral, Southsouth, Southwest, Northwest
173X	Abia, Adamawa, Benue, Borno, Cross River, Ebonyi, Edo, FCT, Jigawa, Kaduna, Kano, Katsina, Kebbi, Lagos, Nasarawa, Niger, Ogun, Osun, Oyo, Rivers, Sokoto, Zamfara states	Southeast, Northeast, Northcentral, Southsouth, Southwest, Northwest
180X	Abia, Adamawa, Bauchi, Benue, Borno, Cross River, Delta, Ebonyi, Enugu, Jigawa, Kaduna, Kano, Katsina, Kebbi, Lagos, Oyo, Rivers, Sokoto, Zamfara states	Southeast, Northeast, Northcentral, Southsouth, Southwest, Northwest
181X	Abia, Bauchi, Cross River, Ebonyi, Enugu, FCT, Imo, Jigawa, Kaduna, Kano, Katsina, Kebbi, Lagos, Oyo, Rivers, Sokoto, Zamfara states	Southeast, Northeast, Northcentral, Southsouth, Southwest, Northwest
184X	Abia, Adamawa, Bauchi, Benue, Borno, Cross River, Ebonyi, Enugu, Ebonyi, Enugu, FCT, Jigawa, Imo, Kaduna, Kano, Katsina, Lagos, Oyo, Sokoto, states	Southeast, Northeast, Northcentral, Southsouth, Southwest, Northwest
202X	Adamawa, Bauchi, Benue, Delta, Ebonyi, Jigawa, Kaduna, Kano, Katsina, Osun, Sokoto, Zamfara states	Southeast, Northeast, Northcentral, Southsouth, Southwest, Northwest
204X	Adamawa, Benue, Cross River, Delta, Ebonyi, Enugu, Kaduna, Kano, Kebbi, Kwara, Lagos, Oyo, Sokoto, Zamfara states	Southeast, Northeast, Northcentral, Southsouth, Southwest, Northwest
L80X	Abia, Adamawa, Benue, Cross River, Delta, Katsina, Kebbi, Kogi, Kwara, Lagos, Ondo, Oyo, Plateau and Sokoto states	Southeast, Northeast, Northcentral, Southsouth, Southwest, Northwest

GP-zone= geopolitical zone

Supplementary Figures

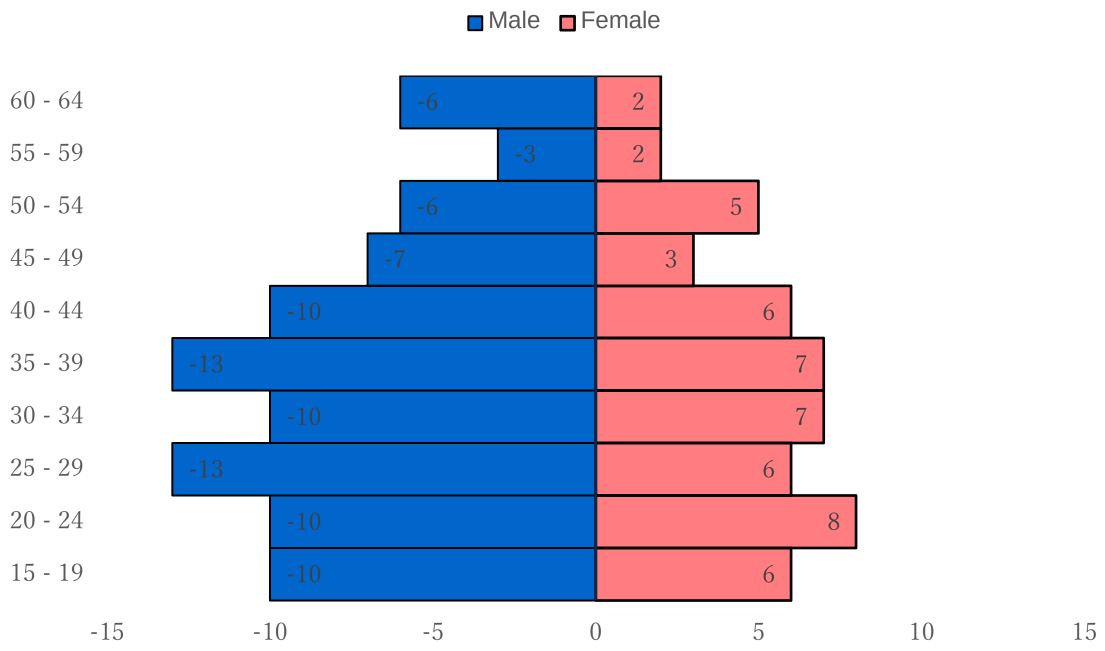

Figure S1: Age-sex population pyramid of HBV^+ in Nigeria, 2024

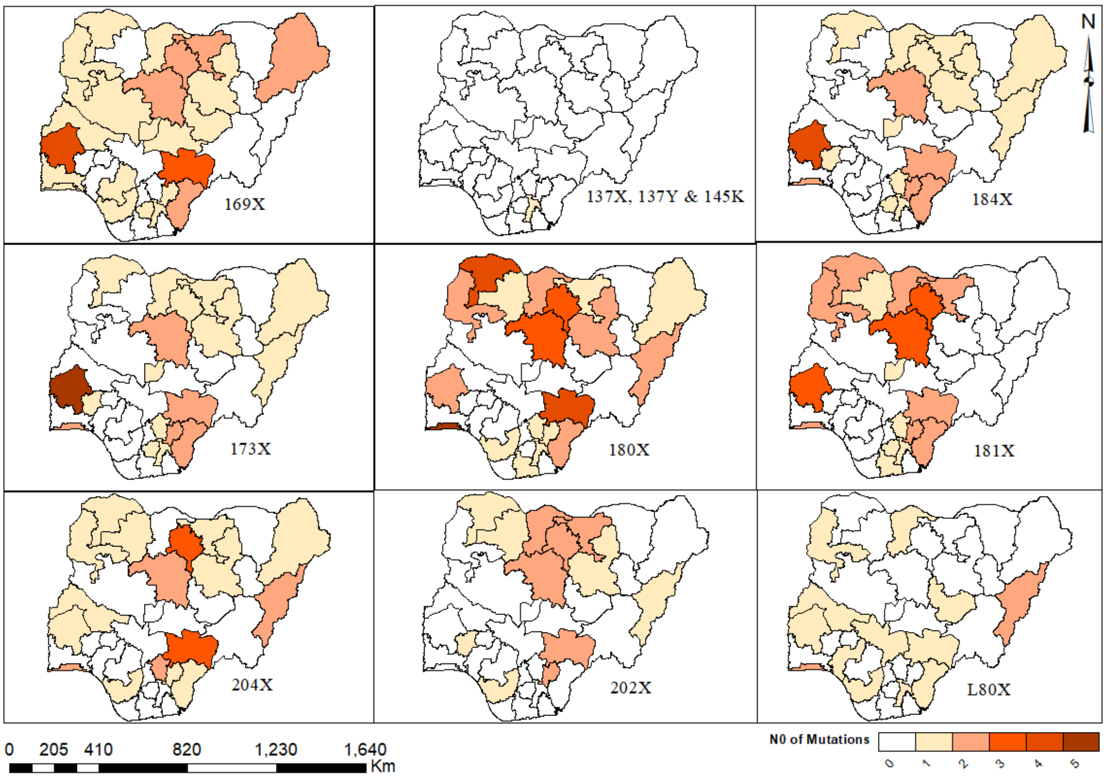

Figure S2: Distribution of HBV variants by states in Nigeria, 2024

References

1. Stuyver L, De Gendt S, Geyt C Van, Zoulim F, Fried M, Schinazi RF, et al. Printed in Great Britain A new genotype of hepatitis B virus : complete genome and phylogenetic relatedness. Journal of General Virology. 2000;81:67–74.
2. Chavan YG, Pawar SR, Wani M, Raut AD, Misra RN. Hepatitis B virus DNA polymerase gene polymorphism based prediction of genotypes in chronic HBV patients from Western India. Afri Health Sci. 2017;17(3).
3. Applied Biosystems. ExoSAP-IT™ Express PCR Product Cleanup Brief Protocol [Internet]. 2024 [cited 2024 Sep 22]. Available from: https://assets.thermofisher.com/TFS-Assets/LSG/manuals/75001b.pdf
4. Lumley SF, Delphin M, Mokaya JF, Tan CCS, Martyn E, Anderson M, et al. A systematic review and meta-analysis of the risk of hepatitis B virus (HBV) resistance in people treated with entecavir or tenofovir. Vol. 174, Journal of Clinical Virology. Elsevier B.V.; 2024.
5. Gao S, Duan ZP, Coffin CS. Clinical relevance of hepatitis B virus variants. Vol. 7, World Journal of Hepatology. Baishideng Publishing Group Co; 2015. p. 1086–96.
6. Yano Y, Azuma T, Hayashi Y. Variations and mutations in the hepatitis B virus genome and their associations with clinical characteristics. World J Hepatol. 2015;7(3):583–92.
7. Lazarevic I. Clinical implications of hepatitis B virus mutations: Recent advances. World J Gastroenterol. 2014;20(24):7653–64.
8. Zhang Q, Liao Y, Cai B, Li Y, Li L, Zhang J, et al. Incidence of natural resistance mutations in naïve chronic hepatitis B patients: A systematic review and meta-analysis. Journal of Gastroenterology and Hepatology (Australia). 2015 Feb 1;30(2):252–61.
9. Aragri M, Alteri C, Battisti A, Di Carlo D, Minichini C, Sagnelli C, et al. Multiple Hepatitis B Virus (HBV) Quasispecies and Immune-Escape Mutations Are Present in HBV Surface Antigen and Reverse Transcriptase of Patients with Acute Hepatitis B. Journal of Infectious Diseases. 2016 Jun 15;213(12):1897–905.
10. Sheldon J, Ramos B, Garcia-Samaniego J, Rios P, Bartholomeusz A, Romero M, et al. Selection of Hepatitis B Virus (HBV) Vaccine Escape Mutants in HBV-Infected and HBV/HIV-Coinfected Patients Failing Antiretroviral Drugs With Anti-HBV Activity Background: Given the overlap between envelope and polymerase [Internet]. Available from: http://journals.lww.com/jaids
11. Caligiuri P, Icardi G, Cerruti R, Bruzzone B. Overview of hepatitis B virus mutations and their implications in the management of infection 2016 Hepatitis B virus: Global view. World J Gastroenterol [Internet]. 2016 [cited 2024 Oct 24];22(1):145–54. Available from:

http://www.wjgnet.com/esps/HelpDesk:http://www.wjgnet.com/esps/helpdesk.aspx
URL:http://www.wjgnet.com/1007-
9327/full/v22/i1/145.htmDOI:http://dx.doi.org/10.3748/wjg.v22.i1.145
